# Total Phenolic Contents and Antioxidant Capacities of Herbal and Tea Infusions

**DOI:** 10.3390/ijms12042112

**Published:** 2011-03-25

**Authors:** Li Fu, Bo-Tao Xu, Ren-You Gan, Yuan Zhang, Xiang-Rong Xu, En-Qin Xia, Hua-Bin Li

**Affiliations:** 1 Guangdong Provincial Key Laboratory of Food, Nutrition and Health, School of Public Health, Sun Yat-Sen University, Guangzhou 510080, China; E-Mails: fulilele@hotmail.com (L.F.); ganry_zsu@yahoo.cn (R.-Y.G.); fly198013@yahoo.com.cn (Y.Z.); enqinxia@163.com (E.-Q.X.); 2 Department of Neurosurgery, Nanfang Hospital, Southern Medical University, Guangzhou 510515, China; E-Mail: xbtfl@yahoo.com; 3 Key Laboratory of Marine Bio-resources Sustainable Utilization, South China Sea Institute of Oceanology, Chinese Academy of Sciences, Guangzhou 510301, China; E-Mail: xuxr@scsio.ac.cn; 4 Liwan District Center for Disease Control and Prevention, Guangzhou 510176, China

**Keywords:** total phenolic content, antioxidant capacity, herbal infusion, tea infusion

## Abstract

In order to supply new information on the antioxidant function of selected beverages for nutritionists and the general public, total phenolic contents of 51 kinds of herbal and tea infusions made in China were measured by the Folin-Ciocalteu method, and their antioxidant capacities were evaluated using ferric reducing antioxidant power (FRAP) and Trolox equivalent antioxidant capacity (TEAC) assays. A significant correlation between FRAP and TEAC values suggested that antioxidant components in these beverages were capable of reducing oxidants and scavenging free radicals. The high correlation between antioxidant capacities and total phenolic contents indicated that phenolic compounds could be one of the main components responsible for antioxidant activities of these beverages. Generally, these beverages had high antioxidant capacities and total phenolic contents, and could be important dietary sources of antioxidant phenolics for prevention of diseases caused by oxidative stress.

## 1. Introduction

Reactive oxygen species may cause a broad spectrum of damage to biological systems, and oxidative stress plays an important role in many chronic and degenerative diseases, such as cardiovascular diseases, cancer, diabetes mellitus and ageing [1–3]. Dietary supplements of antioxidants have become popular to enhance the body’s antioxidant defenses. Natural antioxidants may come from vegetables, fruits and beverages [4–8]. As an important category of phytochemicals, phenolic compounds universally exist in plants. They have attracted increasing attention as potential agents for preventing and treating many oxidative stress-related diseases. At present, there is considerable interest in determining the total phenolic contents and antioxidant capacities of diets. Many herbs and tea have been used to make infusions, and the term “rich in antioxidants” is often used to describe such infusions. However, it usually lacks scientific evidence.

A special kind of herbal infusion is called cool tea (Liang cha in Chinese), which originated from South China. The cool tea is made from some kinds of herbs, and has been drunk as a beverage for hundreds of years. The vendition of cool tea has been from South China to the whole of China, and from China to about 20 countries around the world, such as the United States of America, Canada, United Kingdom, France and Germany. The cool tea has the efficacies of clearing away heat, detoxification, dewetting, moistening lung and stopping thirsty. Similarly, tea has been widely drunk in China for thousands of years. Tea consumption is associated with reduced risks of cardiovascular disease and cancer, and health effects of tea come from its high content of phytochemicals with antioxidant activity [9]. Traditionally, tea is infused only before drinking. Nowadays, a variety of tea infusions have been produced and sold. However, total phenolic contents and antioxidant capacities of herbal and tea infusions made in China have not been evaluated.

The aim of this study was to systematically evaluate total phenolic contents and antioxidant capacities of 51 kinds of herbal and tea infusions made in China, to investigate the relationship between antioxidant capacity and total phenolic content, and to supply new information on the antioxidant function of these beverages for nutritionists and the general public.

## 2. Results and Discussion

### 2.1. Total Phenolic Content of 51 Infusions

Fifty-one kinds of commercial herbal and tea infusions were obtained from markets in Guangzhou, which represent main categories of the infusions made in China (Table 1).

The total phenolic contents of 51 infusions were estimated using the Folin–Ciocalteu method, which relies on the transfer of electrons from phenolic compounds to the Folin–Ciocalteu reagent in alkaline medium, and is a simple and rapid method [10–13]. As shown in Table 2, the total phenolic contents varied from 0.032 ± 0.001 to 1.395 ± 0.068 g gallic acid equivalent (g GAE)/L with the difference of 44-fold, and the mean value was 0.480 g GAE/L for 51 infusions. Ping An Tang li yan cha had the highest total phenolic content (1.395 ±0.068 g GAE/L), but Nian ci an run (chun cui hong se ting zhuang) showed the lowest total phenolic content (0.032 ± 0.001 g GAE/L) among the tested infusions.

For the herbal infusions, the total phenolic contents varied from 0.032 ±0.001 to 1.395 ±0.068 g GAE/L with the difference of 44-fold, and the mean value was 0.410 g GAE/L for the 28 herbal infusions (Table 2). Ping An Tang li yan cha (1.395 ±0.068 g GAE/L) had the highest total phenolic content, followed by Ping An Tang shi gan cha (1.192 ±0.011 g GAE/L), Qing Xin Tang jiang huo wang (1.028 ±0.055 g GAE/L), Qing Xin Tang er shi si wei (1.007 ±0.039 g GAE/L), Qing Xin Tang zhi ke hua tan tang (0.909 ±0.037 g GAE/L), Qing Xin Tang hou zheng tang (0.875 ±0.019 g GAE/L) and Qing Xin Tang gan mao cha (0.844 ± 0.013 g GAE/L). Nian ci an run (chun cui hong se ting zhuang) had the lowest total phenolic content (0.032 ± 0.001 g GAE/L) among the tested herbal infusions.

For the tea infusions, the total phenolic contents varied from 0.253 ± 0.005 to 0.867 ± 0.015 g GAE/L with the difference of 3-fold, and the mean value was 0.565 g GAE/L for the 23 tea infusions (Table 2). Kang Shi Fu muo li mi cha (0.867 ±0.015 g GAE/L) had the highest total phenolic content, followed by Kang Shi Fu muo li qing cha (0.808 ± 0.012 g GAE/L), Tong Yi cha li wang (0.724 ±0.009 g GAE/L), Kang Shi Fu lü cha (0.705 ±0.008 g GAE/L), Ya Tian bing lü cha (0.705 ±0.028 g GAE/L) and Qi Lin hua jian qing yuan (0.696 ±0.025 g GAE/L). Que Chao yuan ye bing hong cha had the lowest total phenolic content (0.253 ± 0.005 g GAE/L) among the tested tea infusions.

When the total phenolic contents of these infusions were compared with those of Serbian white wines and Korean wines reported in the literature [8,14], there was no statistical difference (*p* > 0.05), which indicated that these infusions could contribute the same health benefit as those wines in terms of polyphenols. Taking the negative health effect of alcohol in those wines into account, these infusions would have better health benefits for people [15].

### 2.2. Ferric Reducing Antioxidant Power of 51 Infusions

The ferric reducing antioxidant power (FRAP) assay was used to evaluate antioxidant capacities of the 51 infusions. The FRAP assay is based on the capacity of antioxidants to reduce ferric(III) ions to ferrous(II) ions [16,17], which is a simple and widely used method for the evaluation of antioxidant capacity [18–20]. The FRAP values of 51 infusions are shown in Table 2. In general, these infusions had very high antioxidant capacities. As indicated in Table 2, the FRAP values varied from 0.392 ±0.014 to 30.581 ±1.285 mol Fe(II)/L with the difference of 78-fold, and the mean value was 9.189 mol Fe(II)/L for the 51 infusions. Ping An Tang li yan cha had the highest FRAP value (30.581 ±1.285 mol Fe(II)/L), and Nian ci an run (chun cui hong se ting zhuang) showed the lowest FRAP value (0.392 ±0.014 mol Fe(II)/L) among the tested infusions.

For the herbal infusions, the FRAP values varied from 0.392 ± 0.014 to 30.581 ± 1.285 mol Fe(II)/L with the difference of 78-fold, and the mean value was 6.897 mol Fe(II)/L for 28 herbal infusions (Table 2). Ping An Tang li yan cha (30.581 ± 1.285 mol Fe(II)/L) had the highest FRAP value, followed by Ping An Tang shi gan cha (26.314 ±0.663 mol Fe(II)/L), Qing Xin Tang jiang huo wang (25.454 ±1.175 mol Fe(II)/L), Qing Xin Tang er shi si wei (13.252 ±0.225 mol Fe(II)/L), Qing Xin Tang hou zheng tang (12.490 ± 0.615 mol Fe(II)/L), Qing Xin Tang zhi ke hua tan tang (11.697 ±0.777 mol Fe(II)/L) and Qing Xin Tang gan mao cha (10.382 ±0.845 mol Fe(II)/L). Nian ci an run (chun cui hong se ting zhuang) had the lowest FRAP value (0.392 ±0.014 mol Fe(II)/L) among the tested herbal infusions.

For the tea infusions, the FRAP values varied from 6.454 ±0.280 to 22.724 ±0.758 mol Fe(II)/L with the difference of about 4-fold, and the mean value was 11.979 mol Fe(II)/L for 23 tea infusions (Table 2). Kang Shi Fu muo li mi cha (22.724 ± 0.758 mol Fe(II)/L) had the highest FRAP value, followed by Kang Shi Fu muo li qing cha (20.332 ± 0.543 mol Fe(II)/L), Kang Shi Fu lü cha (18.783 ±0.378 mol Fe(II)/L), Kang Shi Fu wu long ming cha (17.361 ±0.267 mol Fe(II)/L), Kang Shi Fu tie guan ying cha (16.222 ± 0.433 mol Fe(II)/L) and Kang Shi Fu bing lü cha (15.136 ±0.336 mol Fe(II)/L). Tong Yi bing hong cha had the lowest FRAP value (6.454 ±0.280 mol Fe(II)/L) among the tested tea infusions.

**Figure 1.**
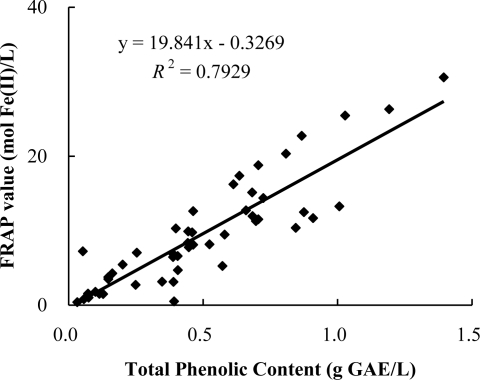
Correlation between total phenolic content and antioxidant capacities measured by the FRAP assay. GAE: gallic acid equivalents.

The correlation between antioxidant capacities and the total phenolic content of the 51 infusions is shown in Figure 1. The result showed a positive linear correlation between the antioxidant capacities and total phenolic content (*R*^2^ = 0.7929), which indicated that phenolic compounds could be one of the main components responsible for antioxidant activities of these beverages.

### 2.3. ABTS^•+^ Radical Scavenging Activity of 51 Infusions

The antioxidant capacities of samples may be influenced by lots of factors, such as test system, and cannot be fully described by one single method. Most natural antioxidants are multifunctional. A reliable antioxidant evaluation protocol requires different antioxidant activity assessments to be performed to take into account various mechanisms of antioxidant action [21]. Therefore, the Trolox equivalent antioxidant capacity (TEAC) assay was used to evaluate free radical scavenging capacities of 51 infusions. The TEAC assay is based on the ability of antioxidants to scavenge ABTS^•+^ radicals. It can measure antioxidant capacities of lipophilic and hydrophilic components in a sample, and is a method usually used for the evaluation of antioxidant capacity [22]. The TEAC values of 51 infusions are given in Table 2. Generally, these infusions had very strong free radical scavenging ability. As seen from Table 2, the TEAC values varied from 0.250 ±0.006 to 19.296 ±0.692 mol Trolox/L with the difference of 77-fold, and the mean value was 5.074 mol Trolox/L for the 51 infusions. Ping An Tang li yan cha had the highest free radical scavenging capacity (19.296 ±0.692 mol Trolox/L), and Nian ci an run (chun cui hong se ting zhuang) showed the lowest free radical scavenging capacity (0.250 ±0.006 mol Trolox/L) among the tested infusions.

For the herbal infusions, the TEAC values varied from 0.250 ± 0.006 to 19.296 ± 0.692 mol Trolox/L with the difference of 77-fold, and the mean value was 3.664 mol Trolox/L for the 28 herbal infusions (Table 2). Ping An Tang li yan cha (19.296 ± 0.692 mol Trolox/L) had the highest free radical scavenging capacity, followed by Ping An Tang shi gan cha (16.269 ± 0.230 mol Trolox/L), Qing Xin Tang zhi ke hua tan tang (6.695 ± 0.114 mol Trolox/L), Qing Xin Tang hou zheng tang (6.499 ±0.046 mol Trolox/L), Qing Xin Tang jiang huo wang (6.474 ±0.019 mol Trolox/L), Qing Xin Tang er shi si wei (6.310 ±0.321 mol Trolox/L) and Qing Xin Tang gan mao cha (6.188 ±0.238 mol Trolox/L). Nian ci an run (chun cui hong se ting zhuang) had the lowest free radical scavenging capacity (0.250 ±0.006 mol Trolox/L) among the tested herbal infusions.

For the tea infusions, the TEAC values varied from 3.815 ±0.087 to 14.020 ±0.324 mol Fe(II)/L with the difference of about 4-fold, and the mean value was 6.791 mol Trolox/L for the 23 tea infusions (Table 2). Kang Shi Fu muo li mi cha (14.020 ± 0.324 mol Trolox/L) had the highest free radical scavenging capacity, followed by Kang Shi Fu muo li qing cha (9.828 ±0.261 mol Trolox/L), Kang Shi Fu lü cha (8.977 ±0.363 mol Trolox/L), Tong Yi cha li wang (8.604 ±0.121 mol Trolox/L), Kang Shi Fu tie guan ying cha (8.361 ± 0.110 mol Trolox/L) and Kang Shi Fu wu long ming cha (8.324 ± 0.069 mol Trolox/L). Qi Lin wu hou hong cha (yuan wei hong cha) had the lowest free radical scavenging capacity (3.815 ±0.087 mol Trolox/L) among the tested tea infusions.

**Figure 2.**
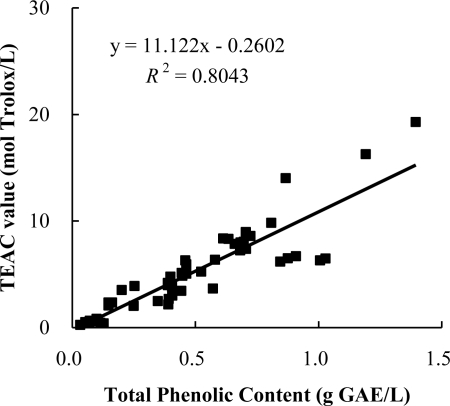
Correlation between total phenolic content and antioxidant capacities measured by the TEAC assay. GAE: Gallic acid equivalents.

**Figure 3.**
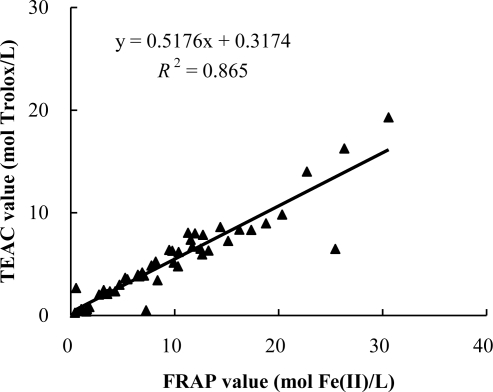
Correlation between total antioxidant capacities measured by the FRAP and TEAC assays.

The correlation between antioxidant capacities and the total phenolic content of the 51 infusions is shown in Figure 2. The result showed a positive linear correlation between the antioxidant capacities and total phenolic content (*R*^2^ = 0.8043), which indicated that phenolic compounds could be one of the main components responsible for antioxidant activities of these infusions. In addition, the correlation between total antioxidant capacities obtained from FRAP and TEAC assays are shown in Figure 3. The results show a positive linear correlation (*R*^2^ = 0.865) between them, which suggested that antioxidant components in these infusions could reduce oxidants (such as ferric ions) and scavenge free radicals. This result is in agreement with those of medicinal plants and wild fruits [23,24]. Maybe, this was because FRAP and TEAC assays are all electron transfer-based methods [25].

Seven herbal infusions and six tea infusions had the strongest antioxidant activities among the 51 infusions based on a combinative consideration of the results obtained by FRAP and TEAC assays as well as the Folin-Ciocalteu method. They are Ping An Tang li yan cha, Ping An Tang shi gan cha, Qing Xin Tang jiang huo wang, Qing Xin Tang er shi si wei, Qing Xin Tang hou zheng tang, Qing Xin Tang zhi ke hua tan tang and Qing Xin Tang gan mao cha as well as Kang Shi Fu muo li mi cha, Kang Shi Fu muo li qing cha, Kang Shi Fu lü cha, Kang Shi Fu wu long ming cha, Kang Shi Fu tie guan ying cha and Tong Yi cha li wang. The main polyphenolic components in these infusions have been identified according to the method reported in the literature [26], and are shown in Table 3. Because of their high antioxidant activities, it could be speculated that these infusions will be beneficial for the diseases caused by oxidative stress.

For total phenolic content, FRAP value and TEAC value, the differences between herbal infusions and tea infusions, between bitter herbal infusions and sweet herbal infusions as well as between green tea infusions and black tea infusions were significant, but the difference between herbal infusions produced by Ping An Tang and those by Qing Xin Tang was not significant (Table 4). In addition, antioxidant capacities of tea infusions, bitter herbal infusions and green tea infusion were higher than those of herbal infusions, sweet herbal infusions and black tea infusion, respectively. Polyphenols are the most important antioxidants in the tea, and catechins are the major phenolic compounds in green tea. Black tea belongs to fermented tea, and its content of catechins was reduced to 20% of that in green tea [27]. Therefore, green tea usually had higher antioxidant capacity than black tea, which resulted in that green tea infusion might have higher antioxidant capacity than black tea infusion. The bitter herbal infusions are usually made from the medicinal plants under the ‘heat-clearing’ category according to the classification of Chinese medicinal plants [12], or those used for prevention and treatment of cold, flu and cough [20], most of which showed the high antioxidant capacities [12,20], while sweet herbal infusions often contain fewer medicinal plants compared with bitter herbal infusions, resulting in lower antioxidant capacities.

## 3. Experimental Section

### 3.1. Chemicals

Gallic acid, 6-hydroxy-2,5,7,8-tetramethylchromane-2-carboxylic acid (Trolox), Folin–Ciocalteu’s phenol reagent, 2,4,6-Tri(2-pyridyl)-s-triazine (TPTZ) and 2,2’-azinobis(3-ethylbenothiazoline-6-sulfonic acid) diammonium salt (ABTS) were obtained from Sigma–Aldrich (St. Louis, MO). Sodium carbonate, potassium persulphate, Iron (III) chloride 6-hydrate, iron (II) sulfate 7-hydrate, acetic acid and sodium acetate were purchased from Tianjing Chemical Factory (Tianjing, China). Hydrochloric acid, ethanol and methanol were obtained from Kelong Chemical Factory (Chengdu, China). All chemicals used in the experiments were of analytical grade, and deionized water was used.

### 3.2. Sample Preparation

Twenty-eight kinds of herbal infusions and twenty-three kinds of tea infusions were bought from local markets (Table 1), which are commercial preparations and in the form of tin with aquatic solution. The samples were kept in the refrigerator at 4 °C until analysis. The various infusions were centrifuged at 3,500 rpm for 30 min, and the resulting supernatants were used for the determination of total phenolic contents and antioxidant capacities.

### 3.3. Determination of Total Phenolic Content

Total phenolic content of the infusion was determined according to the literature [10,28]. Briefly, 0.50 mL of the diluted infusion (a dilution factor of 10-times with water) was added into 2.5 mL of 1:10 diluted Folin–Ciocalteu reagent. After 4 min, 2 mL of saturated sodium carbonate solution (about 75 g/L) was added. The absorbance of the mixture was measured at 760 nm after incubation for 2 h at room temperature. Gallic acid was used as a reference standard and the results were expressed as gram gallic acid equivalent (g GAE)/L of infusion.

### 3.4. Ferric-Reducing Antioxidant Power (FRAP) Assay

The FRAP assay of the infusion was carried out according to the procedure described in the literature [16,17]. Briefly, the FRAP reagent was prepared from sodium acetate buffer (300 mM, pH 3.6), 10 mM TPTZ solution (40 mM HCl as solvent) and 20 mM iron (III) chloride solution in a volume ratio of 10:1:1, respectively. The FRAP reagent was prepared fresh daily and warmed to 37 °C in a water bath before use. One hundred microliters of the diluted infusion was added to 3 mL of the FRAP reagent. After 4 min, the absorbance of the mixture was measured at 593 nm using a Shimadzu UV-2450 ultraviolet-visible spectrophotometer (Japan). The standard curve was constructed using FeSO4 solution, and the results were expressed as mol Fe(II)/L of infusion.

### 3.5. Trolox Equivalent Antioxidant Capacity (TEAC) Assay

The TEAC assay of the infusion was carried out according to the method established in the literature [22]. Briefly, the ABTS^•+^ stock solution was prepared from 7 mM ABTS and 2.45 mM potassium persulfate in a volume ratio of 1:1, and then incubated in the dark for 16 h at room temperature, which should be used within 2 days. The ABTS^+^ working solution was prepared by diluting the stock solution with ethanol to an absorbance of 0.70 ±0.05 at 734 nm. All infusions were aptly diluted to provide 20–80% inhibition of the blank absorbance. One hundred microliters of the diluted infusion was mixed with 3.8 mL ABTS^+^ working solution. After 6 min of incubation at room temperature, the absorbance of the mixture was measured at 734 nm, and the percent of inhibition of absorbance was calculated. Trolox solution was used as a reference standard, and the results were expressed as mol Trolox/L of infusion.

### 3.6. Statistical Analysis

All the experiments were carried out in triplicate, and the results were expressed as mean ± SD (standard deviation). Statistical analysis was performed using SPSS 13.0 and Excel 2003. The *p* value less than 0.05 was considered to be statistically significant.

## 4. Conclusions

The total phenolic contents and antioxidant capacities of 51 kinds of herbal and tea infusions made in China were evaluated. A high correlation between antioxidant capacity and total phenolic content indicated that phenolic compounds could be one of the main components responsible for antioxidant activities of these beverages. A significant correlation between the FRAP value and the TEAC value suggested that antioxidant components in these beverages were capable of reducing oxidants and scavenging free radicals. Generally, these beverages had high total phenolic contents and antioxidant capacities. These beverages could be important dietary sources of antioxidant phenolics for prevention of diseases caused by oxidative stress. This study supplied new information on the antioxidant function of these beverages for consumers, nutritionists and food policy makers. In the future, health effects of these beverages for the consumers should be explored by the epidemiologic method.

## Figures and Tables

**Table 1.** Samples of 51 herbal (H1–H28) and tea (T1–T23) infusions.

**No.**	**Name**	**Name in Chinese**
H1	Ping An Tang xue li ju hua cha	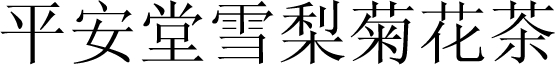
H2	Ping An Tang mao geng zhu zhe shui	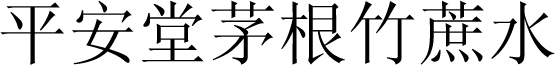
H3	Ping An Tang shen ju cha	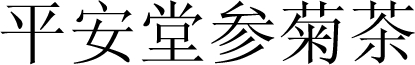
H4	Ping An Tang luo han guo wu hua cha	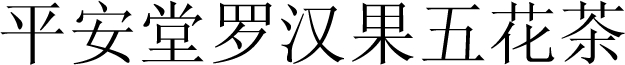
H5	Ping An Tang suan mei tang	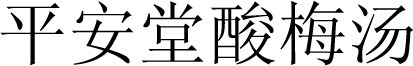
H6	Ping An Tang huo ma ren	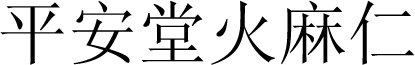
H7*	Ping An Tang li yan cha	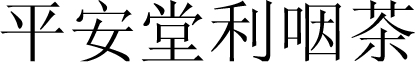
H8*	Ping An Tang shi gan cha	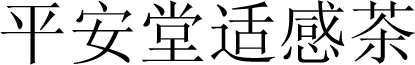
H9	Qing Xin Tang ju hua xue li cha	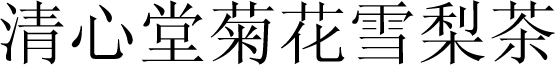
H10	Qing Xin Tang suan mei tang	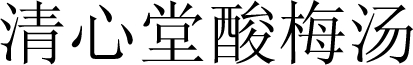
H11	Qing Xin Tang mao geng zhu zhe shui	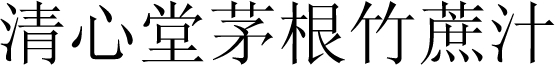
H12	Qing Xin Tang luo han guo wu hua cha	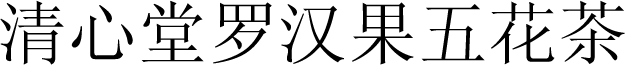
H13*	Qing Xin Tang gan mao cha	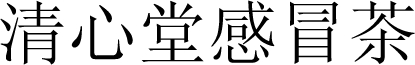
H14*	Qing Xin Tang zhi ke hua tan tang	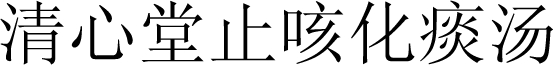
H15*	Qing Xin Tang hou zheng tang	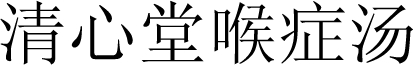
H16*	Qing Xin Tang jiang huo wang	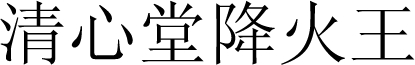
H17*	Qing Xin Tang er shi si wei	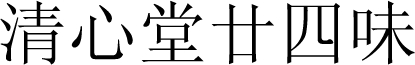
H18	Deng lao liang cha	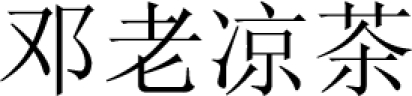
H19	Wang lao ji (ting zhuang)	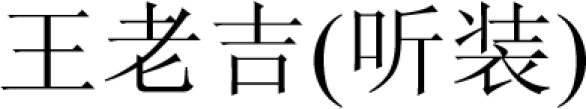
H20	Wang lao ji (he zhuang)	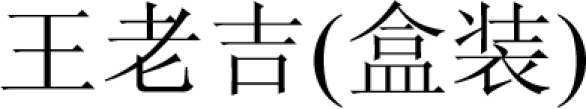
H21	Qing liang cha (he zhuang)	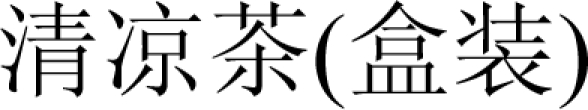
H22	Nian ci an run (qing xing lü se ting zhuang)	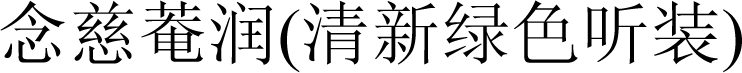
H23	Nian ci an run (chun cui hong se ting zhuang)	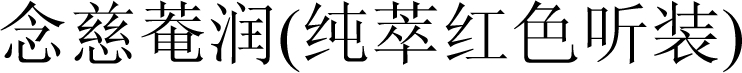
H24	Bao Qing Tang xue li ju hua cha	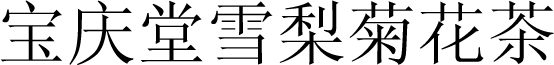
H25	Pan Gao Shou liang cha	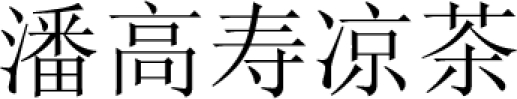
H26*	Er shi si wei	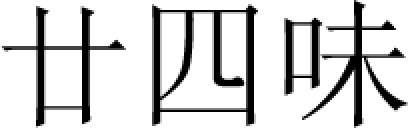
H27	Bai Yun Shan liang cha	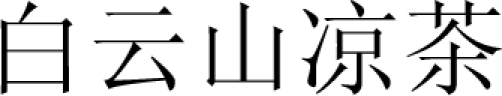
H28	Ben cao mi liang cha	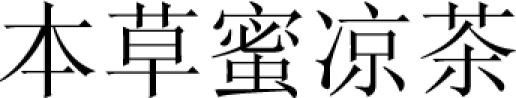
T1	Kang Shi Fu bing lü cha	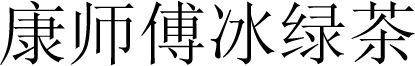
T2	Kang Shi Fu bing hong cha	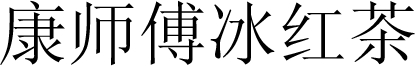
T3	Kang Shi Fu jing liang bing lü cha	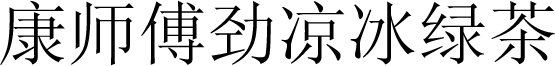
T4	Kang Shi Fu jing liang bing hong cha	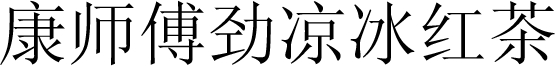
T5	Kang Shi Fu muo li mi cha	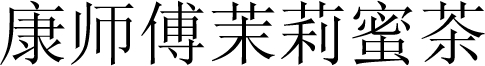
T6	Kang Shi Fu muo li qing cha	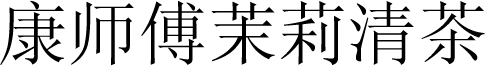
T7	Kang Shi Fu lü cha	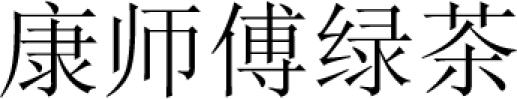
T8	Kang Shi Fu tie guan ying cha	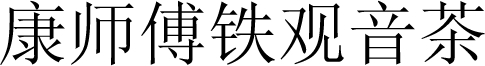
T9	Kang Shi Fu wu long ming cha	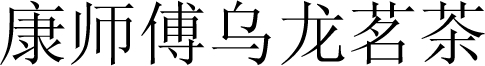
T10	Que Chao yuan ye bing hong cha	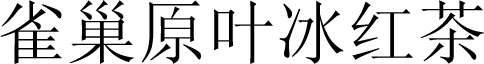
T11	Ya Tian bing lü cha	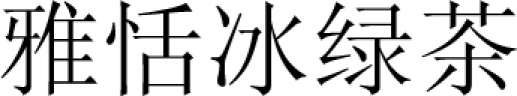
T12	Ya Tian bing hong cha	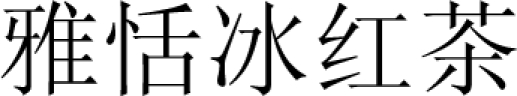
T13	Tong Yi you ji lü cha	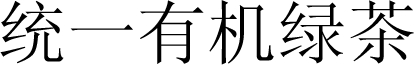
T14	Tong Yi cha li wang	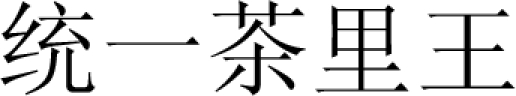
T15	Tong Yi bing hong cha	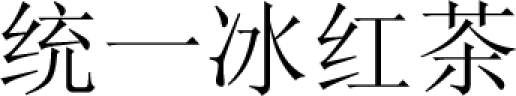
T16	Tong Yi bing lü cha	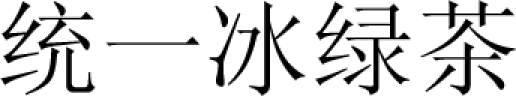
T17	Tong Yi lü cha	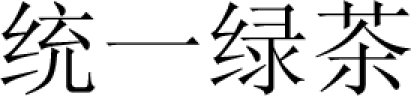
T18	Qi Lin wu hou hong cha (ning meng cha)	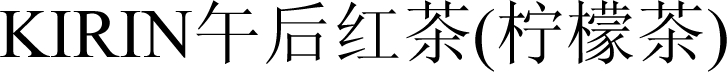
T19	Qi Lin wu hou hong cha (yuan wei hong cha)	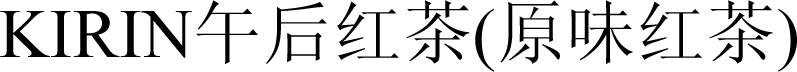
T20	Qi Lin wu hou hong cha (bing jing ning meng)	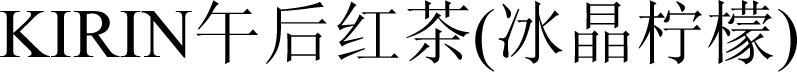
T21	Qi Lin cha wu	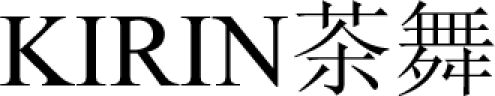
T22	Qi Lin hua jian qing yuan	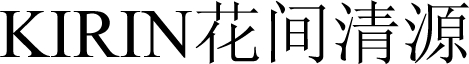
T23	Qi Lin sheng cha	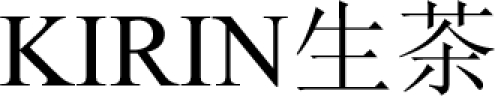

* For herbal infusions, No. with * were bitter herbal teas, and the others were sweet herbal teas.

**Table 2.** The antioxidant capacities and total phenolic contents of 51 herbal and tea infusions.

**No.**	**FRAP values**	**TEAC values**	**Total phenolic contents**
H1	4.687 ±0.208	2.988 ±0.177	0.406 ±0.014
H2	1.003 ±0.024	0.613 ±0.015	0.074 ±0.003
H3	7.234 ±0.212	0.504 ±0.007	0.053 ±0.001
H4	5.452 ±0.088	3.529 ±0.060	0.201 ±0.009
H5	0.506 ±0.011	2.673 ±0.094	0.392 ±0.009
H6	1.504 ±0.101	0.386 ±0.007	0.128 ±0.005
H7	30.581 ±1.285	19.296 ±0.692	1.395 ±0.068
H8	26.314 ±0.663	16.269 ±0.230	1.192 ±0.011
H9	2.722 ±0.116	2.040 ±0.085	0.249 ±0.004
H10	3.114 ±0.168	2.176 ±0.064	0.390 ±0.003
H11	3.162 ±0.116	2.480 ±0.074	0.347 ±0.008
H12	5.246 ±0.266	3.669 ±0.036	0.572 ±0.005
H13	10.382 ±0.845	6.188 ±0.238	0.844 ±0.013
H14	11.697 ±0.777	6.695 ±0.114	0.909 ±0.037
H15	12.490 ±0.615	6.499 ±0.046	0.875 ±0.019
H16	25.454 ±1.175	6.474 ±0.019	1.028 ±0.055
H17	13.252 ±0.225	6.310 ±0.321	1.007 ±0.039
H18	8.341 ±0.322	3.438 ±0.076	0.443 ±0.013
H19	3.508 ±0.039	2.083 ±0.085	0.147 ±0.002
H20	3.764 ±0.151	2.348 ±0.013	0.148 ±0.001
H21	1.550 ±0.040	0.637 ±0.004	0.072 ±0.001
H22	0.812 ±0.016	0.428 ±0.016	0.056 ±0.001
H23	0.392 ±0.014	0.250 ±0.006	0.032 ±0.001
H24	1.218 ±0.028	0.445 ±0.018	0.070 ±0.001
H25	1.159 ±0.020	0.537 ±0.013	0.068 ±0.001
H26	1.785 ±0.055	0.825 ±0.013	0.099 ±0.001
H27	1.510 ±0.020	0.446 ±0.009	0.114 ±0.004
H28	4.279 ±0.082	2.351 ±0.093	0.162 ±0.005
T1	15.136 ±0.336	7.251 ±0.129	0.682 ±0.009
T2	9.910 ±0.125	5.139 ±0.201	0.445 ±0.007
T3	12.628 ±0.311	5.931 ±0.172	0.463 ±0.002
T4	10.308 ±0.538	4.779 ±0.217	0.399 ±0.008
T5	22.724 ±0.758	14.020 ±0.324	0.867 ±0.015
T6	20.332 ±0.543	9.828 ±0.261	0.808 ±0.012
T7	18.783 ± 0.378	8.977 ± 0.363	0.705 ± 0.008
T8	16.222 ± 0.433	8.361 ± 0.110	0.613 ± 0.008
T9	17.361 ± 0.267	8.324 ± 0.069	0.634 ± 0.015
T10	7.047 ± 0.296	3.896 ± 0.038	0.253 ± 0.005
T11	11.538 ± 0.523	7.376 ± 0.208	0.705 ± 0.028
T12	6.874 ± 0.336	4.187 ± 0.120	0.388 ± 0.009
T13	9.766 ± 0.536	6.308 ± 0.217	0.459 ± 0.021
T14	14.383 ± 0.410	8.604 ± 0.121	0.724 ± 0.009
T15	6.454 ± 0.280	3.970 ± 0.168	0.388 ± 0.018
T16	8.121 ± 0.406	5.062 ± 0.068	0.463 ± 0.014
T17	12.722 ± 0.698	7.853 ± 0.126	0.660 ± 0.016
T18	8.164 ± 0.591	5.239 ± 0.282	0.523 ± 0.015
T19	6.595 ± 0.412	3.815 ± 0.087	0.405 ± 0.017
T20	7.764 ± 0.316	4.854 ± 0.104	0.447 ± 0.009
T21	9.461 ± 0.284	6.371 ± 0.202	0.580 ± 0.014
T22	11.286 ± 0.561	8.057 ± 0.239	0.696 ± 0.025
T23	11.943 ± 0.270	7.987 ± 0.086	0.683 ± 0.011

**Table 3.** Main polyphenolic components in herbal and tea infusions showing the highest phenolic contents and antioxidant activities.

**Name**	**No.**	**Main polyphenolic components**
Ping An Tang li yan cha	H7	gallic acid, gallocatechin, β-resorcylic acid, luteolin-o-diglucose, o-coumaric acid, hesperetin-7-o-rutinoside, apigenin, kaempferol
Ping An Tang shi gan cha	H8	gallic acid, gallocatechin, chlorogenic acid, luteolin-o-diglucose, o-coumaric acid, myricetin, apigenin-o-glucose, daidzein, chalcone
Qing Xin Tang jiang huo wang	H16	gallic acide, β-resorcylic acid, chlorogenic acid, luteolin-o-diglucose, daidzein, quercetin, kaempferol, chalcone
Qing Xin Tang er shi si wei	H17	gallocatechin, β-resorcylic acid, chlorogenic acid, luteolin-o-diglucose, quercetin, kaempferol, chalcone
Qing Xin Tang hou zheng tang	H15	β-resorcylic acid, chlorogenic acid, luteolin-o-diglucose, apigenin-o-glucose, daidzein, quercetin, luteotin, kaempferol, galangin
Qing Xin Tang zhi ke hua tan tang	H14	β-resorcylic acid, chlorogenic acid, luteolin-o-diglucose, apigenin-o-glucose, daidzein, quercetin, kaempferol, chalcone
Qing Xin Tang gan mao cha	H13	gallocatechin, β-resorcylic acid, chlorogenic acid, luteolin-o-diglucose, myricetin, quercetin, kaempferol, galangin
Kang Shi Fu muo li mi cha	T5	gallic acid, gallocatechin, protocatechuic acid, caffeic acid, epigallocatechin gallate, p-coumatic acid, kaempferol, galangin
Kang Shi Fu muo li qing cha	T6	gallic acid, gallocatechin, protocatechuic acid, caffeic acid, epigallocatechin gallate, p-coumatic acid
Kang Shi Fu lü cha	T7	gallic acid, gallocatechin, protocatechuic acid, chlorogenic acid, caffeic acid, epigallocatechin gallate, p-coumatic acid
Kang Shi Fu wu long ming cha	T9	gallic acid, gallocatechin, protocatechuic acid, chlorogenic acid, caffeic acid, epigallocatechin gallate, p-coumatic acid, thea flavin
Kang Shi Fu tie guan ying cha	T8	gallic acid, protocatechuic acid, chlorogenic acid caffeic acid, epigallocatechin gallate, p-coumatic acid, thea flavin
Tong Yi cha li wang	T14	gallic acid, gallocatechin, protocatechuic acid, chlorogenic acid, caffeic acid, epigallocatechin gallate, p-coumatic acid

**Table 4.** Comparison of different infusions.

**Parameter**	**FRAP values**	**TEAC values**	**Total phenolic contents**
Herbal infusions *vs.* Tea infusions	P < 0.001	P < 0.001	P = 0.011 < 0.05
Bitter herbal infusions *vs.* Sweet herbal infusions	P < 0.001	P < 0.001	P = 0.001 < 0.05
Herbal infusions of Ping An Tang *vs.* That of Qing Xin Tang	P = 0.501 > 0.05	P = 0.386 > 0.05	P = 0.248 > 0.05
Green tea infusions *vs*. Black tea infusions	P = 0.001 < 0.05	P = 0.001 < 0.05	P < 0.001
